# Novel Lesions of Bones and Joints Associated with Chikungunya Virus Infection in Two Mouse Models of Disease: New Insights into Disease Pathogenesis

**DOI:** 10.1371/journal.pone.0155243

**Published:** 2016-05-16

**Authors:** Brad A. Goupil, Margaret A. McNulty, Matthew J. Martin, Michael K. McCracken, Rebecca C. Christofferson, Christopher N. Mores

**Affiliations:** 1 Department of Pathobiological Sciences, Louisiana State University School of Veterinary Medicine, Skip Bertman Drive, Baton Rouge, Louisiana, United States of America; 2 Department of Comparative Biomedical Sciences, Louisiana State University School of Veterinary Medicine, Skip Bertman Drive, Baton Rouge, Louisiana, United States of America; CEA, FRANCE

## Abstract

Chikungunya virus is an arbovirus spread predominantly by *Aedes aegypti* and *Ae*. *albopictus* mosquitoes, and causes debilitating arthralgia and arthritis. While these are common manifestations during acute infection and it has been suggested they can recur in patients chronically, gaps in knowledge regarding the pathogenesis still exist. Two established mouse models were utilized (adult IRF 3/7 ^-/- -/-^ and wild-type C57BL/6J mice) to evaluate disease manifestations in bones and joints at various timepoints. Novel lesions in C57BL/6J mice consisted of periostitis (91%) and foci of cartilage of necrosis (50% of mice at 21 DPI). Additionally, at 21 DPI, 50% and 75% of mice exhibited periosteal bone proliferation affecting the metatarsal bones, apparent via histology and μCT, respectively. μCT analysis did not reveal any alterations in trabecular bone volume measurements in C57BL/6J mice. Novel lesions demonstrated in IRF 3/7 ^-/- -/-^ mice at 5 DPI included focal regions of cartilage necrosis (20%), periosteal necrosis (66%), and multifocal ischemic bone marrow necrosis (100%). Contralateral feet in 100% of mice of both strains had similar, though milder lesions. Additionally, comparison of control IRF 3/7 ^-/- -/-^ and wild-type C57BL/6J mice demonstrated differences in cortical bone. These experiments demonstrate novel manifestations of disease similar to those occurring in humans, adding insight into disease pathogenesis, and representing new potential targets for therapeutic interventions. Additionally, results demonstrate the utility of μCT in studies of bone and joint pathology and illustrate differences in bone dynamics between mouse strains.

## Introduction

Chikungunya virus (CHIKV) is an alphavirus in the *Togaviridae* family. The word ‘chikungunya’ is roughly translated to “that which bends up” or “to walk bent over” from the Makonde language in Tanzania and is so named because of the severe incapacitating arthralgia that occurs with infection [1,2]. CHIKV is an enveloped, positive sense RNA arbovirus spread predominantly by *Aedes aegypti* and *Ae*. *albopictus* mosquitoes [3–5].

Historically, the geographical distribution of CHIKV has been restricted to tropical and subtropical regions throughout Africa and Asia, particularly the Indian subcontinent. There are three currently recognized genotypes of virus endemic in these areas, defined as the Eastern/Central/South African, West African, and Asian genotypes [6]. Due to the expanding range of the vector *Ae*. *albopictus* and the continually increasing ease of international travel and commerce, CHIKV has recently extended into temperate regions [1,7–10]. Most recently, beginning in October of 2013, the virus has emerged and rapidly spread throughout the Caribbean and the Americas, resulting in more than 1.6 million suspected cases and 270 deaths as of October 15, 2015 [11,12]. It has been determined that the strain circulating in this region belongs to the Asian genotype [13].

Approximately 95% of people that are infected with CHIKV will become symptomatic, which consists predominantly of high fever, rash, and debilitating arthralgia [2,14–16]. While in the majority of cases symptoms resolve approximately two weeks after onset, some patients can develop ongoing symptoms of arthralgia lasting months to years after the initial infection [1,16–22]. Most commonly, there is symmetrical involvement of distal joints including those of the ankles, wrists, and digits, though any joint can be affected [2,14,20,23]. While there are numerous reports of clinical workups on patients suffering from chronic CHIKV-associated arthralgia, the mechanisms involved in the development of disease and their association with initial viral infection are unclear [2,13,16,20,23–26]. However, some reports suggests that chronic CHIKV-associated arthritis is an erosive and inflammatory arthritis with similarities to rheumatoid arthritis [23,27]. Diagnostic imaging techniques such as radiography and MRI have demonstrated evidence of loss of articular cartilage and bony erosions in affected joints of patients with histories of CHIKV infection [1,15,23,24]. However, it should be noted that in these cases, these chronic joint manifestations cannot be unequivocally attributed to previous infection with CHIKV, as other concurrent joint diseases such as rheumatoid arthritis or osteoarthritis cannot be ruled out.

The arthralgia and/or arthritis associated with CHIKV infection represents a significant component of the clinical picture of both acute and chronic disease, though there are still gaps in knowledge regarding the pathogenesis of these manifestations. Current hypotheses for chronic disease include viral or antigen persistence within tissues of the joint (e.g. synovium) resulting in ongoing direct damage from viral infection, replication and/or immune-mediated tissue destruction, or induction of a post-infection autoimmune condition [3,25,28–30]. The challenge in elucidating these mechanisms of bone and joint disease is that animal models of chronic CHIKV-associated arthralgia and/or arthritis are lacking, and existing models of acute disease do not necessarily replicate all aspects of human disease manifestations [5,31,32]. While existing models can recapitulate some aspects of disease, involvement of the articular cartilage and bone in particular have rarely been definitively demonstrated [33,34]. In addition to the bone and joint manifestations of disease, in rare cases, CHIKV infection can result in more severe disease manifestations, including mortalities [5,35–38].

There are currently several existing animal models including mice and non-human primates that are considered appropriate for evaluation of CHIKV-associated disease as experimental models [32,35,39]. Two models that most closely replicate consistent joint involvement associated with CHIKV infection include the IRF 3/7 ^-/- -/-^ mice, which have a deficient type 1 interferon response, and adult, wild-type C57BL/6 mice [40–42]. The IRF 3/7 ^-/- -/-^ mice develop a fatal form of disease, similar to that seen in some of the more severe manifestations of disease in people [41,42], while the C57BL/6 mice develop a more protracted form of disease, similar to that seen in the majority of human cases [40]. Both models have been shown to develop significant viremia following viral inoculation in the dorsal or ventral surface of the hind foot, exhibit evidence of some myositis, synovitis, and tendinitis associated with infection, and CHIKV has been demonstrated locally within the tissues of the foot [40–42]. While these two mouse models develop consistent CHIKV-associated disease, some of the manifestations of joint disease seen in humans, including damage to the articular cartilage, alterations of bone including bony lysis, have rarely, if ever been described. Additionally, none of these models have demonstrated utility for use in chronic CHIKV-associated disease studies.

By utilizing these two established animal models of disease, this study aimed to 1) demonstrate, describe and evaluate pathologic findings in both articular cartilage and bone associated with CHIKV infection, 2) examine these aspects of disease manifestations over time at various points throughout the disease course, and 3) evaluate these models for potential use in chronic studies of CHIKV-associated arthralgia/arthritis. We suspect that a thorough evaluation of bone and joint pathology associated with CHIKV infection will reveal previously unrecognized lesions in these existing mouse models. We hypothesize that CHIKV infection will result in degeneration of articular cartilage and osteoclastic bone resorption associated with synovitis.

## Materials and Methods

### Virus

A Southeast Asian strain of CHIKV (SVO 476–96) was utilized for this project and was a generous gift from the World Reference Center for Emerging Viruses and Arboviruses at the University of Texas Medical Branch. The isolate was originally obtained from a human sample in Northeast Thailand in 1996 and was passaged twice in LLC-MK2 cells and once in Vero cells prior to being obtained by our laboratory. The virus was passaged one additional time in Vero cells prior to utilization in this project.

### Ethics Statement

Housing of animals utilized in this study and the methods employed during animal experimental procedures were reviewed and approved by the Louisiana State University (LSU) Institutional Animal Care and Use Committee (IACUC) which complies with the guidelines stated in the National Institutes of Health’s (NIH) Guide for the Care and Use of Laboratory Animals. These experiments were conducted under the approved LSU IACUC protocol #15–005 (approved in 2015).

### Animal models

Two established mouse models of CHIKV infection were utilized: IRF 3/7 ^-/- -/-^ mice on a C57BL/6 background (≥ 8 weeks of age; males and females) and C57BL/6J mice (≥ 8 weeks of age; females only). C57BL/6J mice were obtained from a distributor (The Jackson Laboratory, https://www.jax.org/). The IRF 3/7 ^-/- -/-^ mice were originally obtained as a generous gift from Dr. Michael Diamond (Washington University, St. Louis, MO) with permission from Dr. T. Taniguchi (University of Tokyo, Tokyo, Japan). A breeding colony was subsequently established and maintained in the BSL3 laboratory at the Louisiana State University (LSU) School of Veterinary Medicine (SVM).

Mice were housed in groups of 4 or 5 in micro-isolator cages in the BSL3 laboratory of the LSU SVM. Mice were provided water and a standard commercial mouse food (LabDiet, Land O’ Lakes, Inc., St. Louis, MO) ad libitum throughout the experiments. Prior to infection, mice were initially anesthetized with isoflurane followed by intraperitoneal injection of a ketamine (40 mg/kg) and xylazine (2 mg/kg) solution. Subsequently, mice were intradermally (IRF 3/7 ^-/- -/-^ mice) or subcutaneously (C57BL/6J mice) injected in the caudoventral aspect of the hind foot near the tarsal joint with 2x10^4^ PFU of virus. As negative controls, age and sex-matched mice were injected with a similar volume (10μl) of sterile PBS. Mice were then euthanized at various timepoints throughout the disease course (1, 2, 4, 5, 6, 7 days post infection [DPI] for the IRF 3/7 ^-/- -/-^ mice and 7, 14, 21 DPI for the C57BL/6J mice). During experiments, animals were monitored a minimum of once (C57BL/6J mice) or twice (IRF-3/7^-/- -/-^ mice) daily for clinical signs associated with illness, including lethargy, abnormal posture, joint swelling, lameness, limb gnawing, and loss of body weight. If at any point during the experiments, mice were determined to be in significant distress or exhibited a loss of 20% or greater of initial body weight, they were immediately euthanized to reduce pain and suffering. At 5 DPI, 4 of the IRF 3/7 ^-/- -/-^ mice were moderately lethargic and were subsequently euthanized at that time. Due to the rapid progression of the fatal disease that occurs in the IRF 3/7 ^-/- -/-^ mice, 6 mice died during the experiment, though, with the exception of foot swelling, no additional clinical signs were observed in these mice prior to their death (4 mice at 5 DPI, 1 mouse at 6 DPI, and 1 mouse at 7 DPI). Mice were euthanized via CO_2_ asphyxiation followed by cervical dislocation. All experiments were performed in accordance with LSU IACUC guidelines (protocol #15–005).

### Microcomputed tomography (μCT)

Intact hindlimbs were collected from mice and scanned using μCT. Two regions of interest (ROI) scanned for each mouse included: 1) the stifle joint extending from approximately mid-shaft of the femur to mid-shaft of the tibia and fibula, and 2) the distal hindlimb extending from the proximal aspect of the tuber calcanei to the distal phalanges. Specimens were placed in holders for scanning by μCT (Scanco model 40; Scanco Medical AG, Basserdorf, Switzerland). Samples were scanned in a transverse plane in 70% ETOH at 55kV, 0.3-second integration time, with a 16μm voxel size in plane and a 16μm slice thickness. To evaluate differences in bone morphology within age-matched IRF 3/7 ^-/- -/-^ mice (n = 11) compared to wild-type C57BL/6J controls (n = 9), the humeri were dissected free from surrounding soft tissue and scanned in 70% ETOH at 55kV, 0.3-second integration time, with a 10μm voxel size in plane and a 10μm slice thickness.

To evaluate trabecular bone morphology secondary to CHIKV infection within the stifle joint region, two ROIs were evaluated: 1) the proximal epiphysis of the tibia extending from the subchondral bone to the proximal physis as has been previously evaluated [43], and 2) the distal metaphysis of the femur, extending from approximately mid-shaft to the distal physis, which is considered the appropriate location to evaluate trabecular bone morphology [44]. The proximal 30% of the humerus, extending distally from the proximal physis, was the ROI used to evaluate changes in trabecular morphology within the IRF 3/7 ^-/- -/-^ strain compared to wild-type C57BL/6J controls, and a region at midshaft was used to evaluate cortical bone.

The proper thresholds for 3D reconstructions to qualitatively evaluate overall bone morphology, in addition to appropriate thresholds to quantitatively evaluate trabecular bone, were tested and the same thresholds were used throughout the study for all ROIs. For trabecular bone, bone volume (BV), total volume (TV), BV/TV, tissue density, apparent density, trabecular number (Tb.N), trabecular thickness (Tb.Th), trabecular spacing (Tb.Sp), connectivity density (Conn.D), structural model index (SMI), bone surface (BS), and degree of anisotropy (DA) were evaluated. For cortical bone, cortical area (Ct.Ar), thickness (Ct.Th), and porosity (Ct.Po), total cross-sectional area (Tt.Ar), and Ct.Ar/Tt.Ar were evaluated. Established procedures and nomenclature were used for all variables [44].

### Histopathology

Immediately following euthanasia, entire mice were fixed in 10% neutral buffered formalin for a minimum of 48 hours. Following fixation and after evaluation via μCT, the entire hind feet of mice (ipsilateral and contralateral) were sagittally bisected and decalcified in 10% EDTA for 21–28 days. Samples were then routinely processed for histopathologic evaluation. Briefly, samples were dehydrated in increasing concentrations of ethanol and embedded in paraffin wax. Subsequently, 4–5 μm thick sections were adhered to positively charged glass slides. Sections were routinely stained with hematoxylin and eosin (H&E) for evaluation. Standard Masson’s trichrome stains were performed on select slides. All histologic evaluations were performed by a board-certified veterinary anatomic pathologist (BAG). All slides were evaluated using a Nikon Eclipse Ni-U upright microscope (Nikon Instruments Inc., Melville, New York) and digital images were obtained using an attached Olympus DP72 microscope digital camera (Olympus Corporation, Tokyo, Japan).

## Results

### C57BL/6 mice

**Fig 1:**
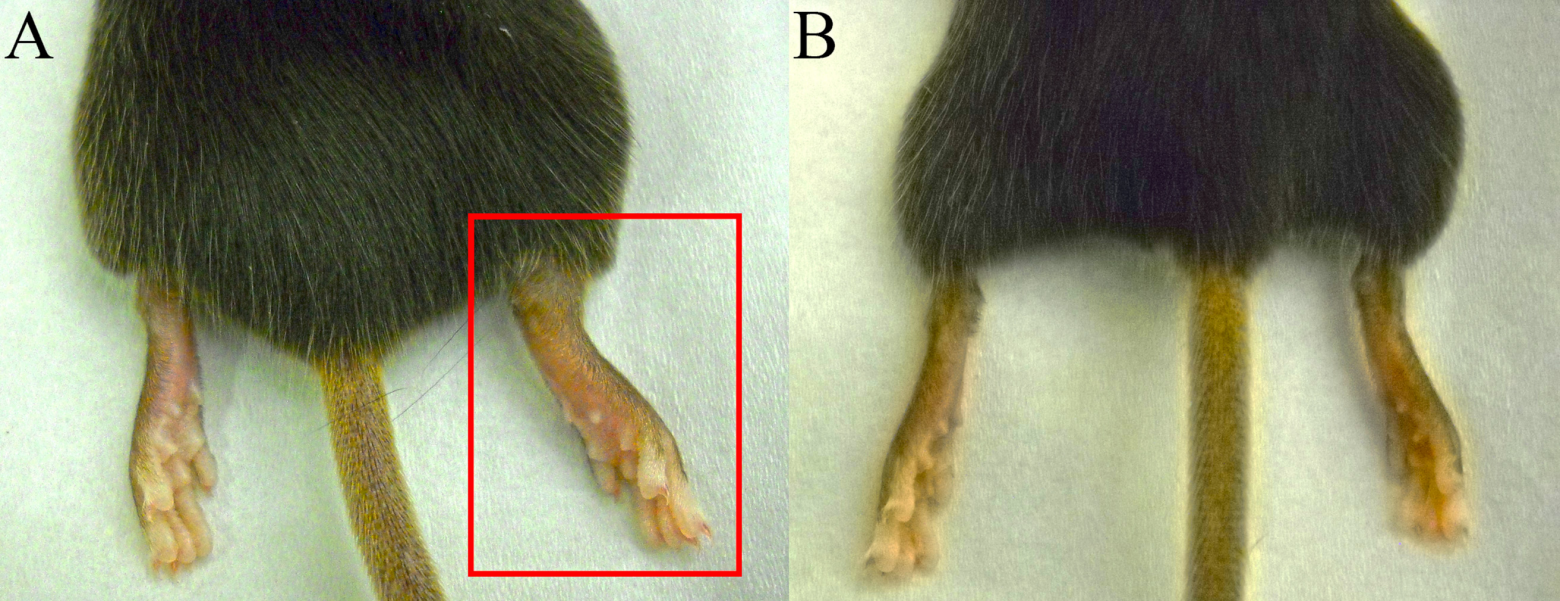
C57BL/6 mice sacrificed at 7 DPI. (A) CHIKV inoculated mouse demonstrating significant swelling of the inoculated foot (right foot; red box). (B) PBS sham inoculated mouse demonstrating no swelling associated with inoculation (right foot).

At 7 DPI, all virally infected mice exhibited moderate to marked swelling of the inoculated feet (Fig 1). This swelling had resolved by 14 DPI in all mice. These findings are similar to those previously reported in this mouse model [40].

**Fig 2:**
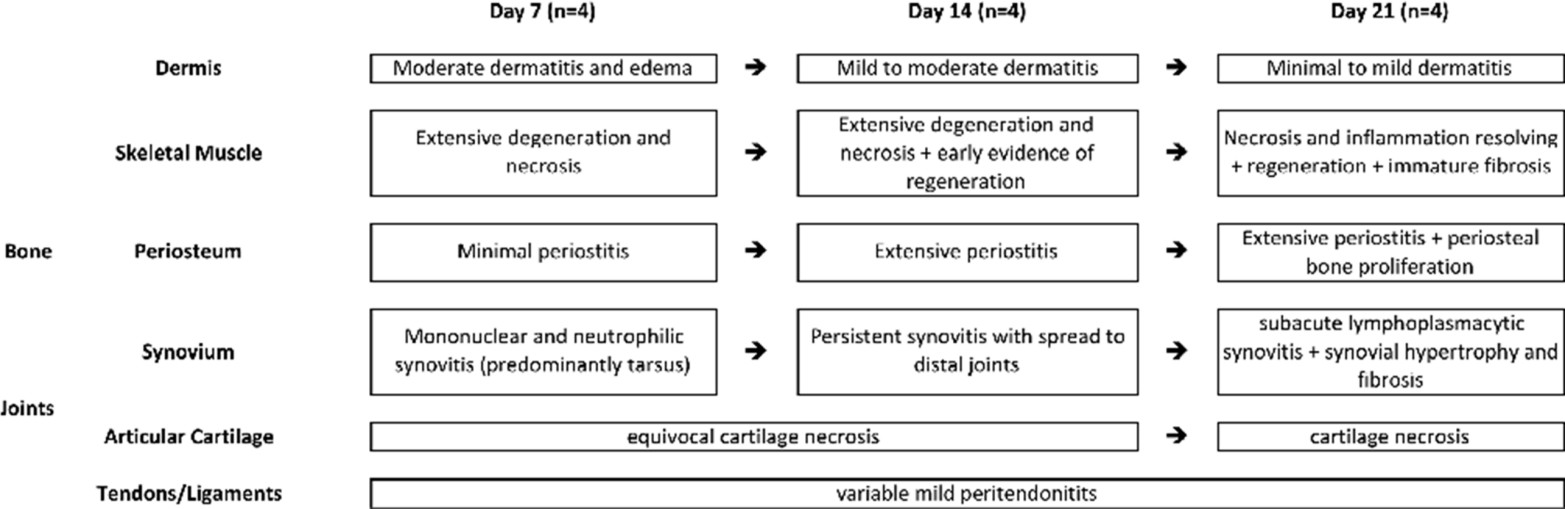
Progression of lesions in C57BL/6J mice infected with chikungunya virus.

Initial histopathologic evaluation of some aspects of CHIKV infection in the feet of adult C57Bl/6 mice has been previously reported [40]. Similar findings were observed in the current experiment, including moderate mononuclear inflammation in the deep dermis and subcutis, extensive myocyte necrosis and inflammation, tendonitis, and synovitis. A more detailed histologic evaluation in the current experiment demonstrated additional novel lesions associated with CHIKV infection. An overview of the various lesions and their time courses are presented in Fig 2.

In 100% of mice, the predominant change in the skeletal muscle consisted of acute myofiber degeneration and necrosis accompanied by minimal cellular infiltrates consisting predominantly of macrophages and fewer neutrophils. Joint lesions primarily affected the tarsal joints in 100% of mice, and consisted of loss of the lining synoviocytes of the intima and infiltration of the subintima by small numbers of mononuclear cells and neutrophils. There were rare regions of equivocal cartilage necrosis in few mice, though no definitive foci were apparent. Affecting the metatarsal bones of 3 of 4 infected mice (75%), there were occasional areas of minimal to mild expansion and hypercellularity of the periosteum (periostitis). Rarely, within the tibia and metatarsal bones of 1 mouse (25%), there were small areas of bone marrow edema characterized by accumulation of variably sized pools of pale eosinophilic, homogenous material.

At 14 DPI, the necrosis and inflammatory component within the skeletal muscle was similar to that observed at 7 DPI, however, there was also evidence of myofiber regeneration characterized by the presence of large myocytes containing basophilic cytoplasm and internalized nuclei exhibiting nuclear rowing. More distal joints including the tarsometatarsal, metatarsophalangeal and interphalangeal joints were also similarly affected in all mice. Similarly to 7 DPI, there were rare regions of equivocal cartilage necrosis in few mice, though no definitive foci were apparent. Four of 4 mice (100%) had consistent mild periostitis affecting the majority of the length of the metatarsal bones. Areas exhibiting more significant periostitis were often immediately adjacent to more extensive regions of necrosis and inflammation within the skeletal muscle. Bone marrow edema was not observed in any mice at this timepoint.

**Fig 3:**
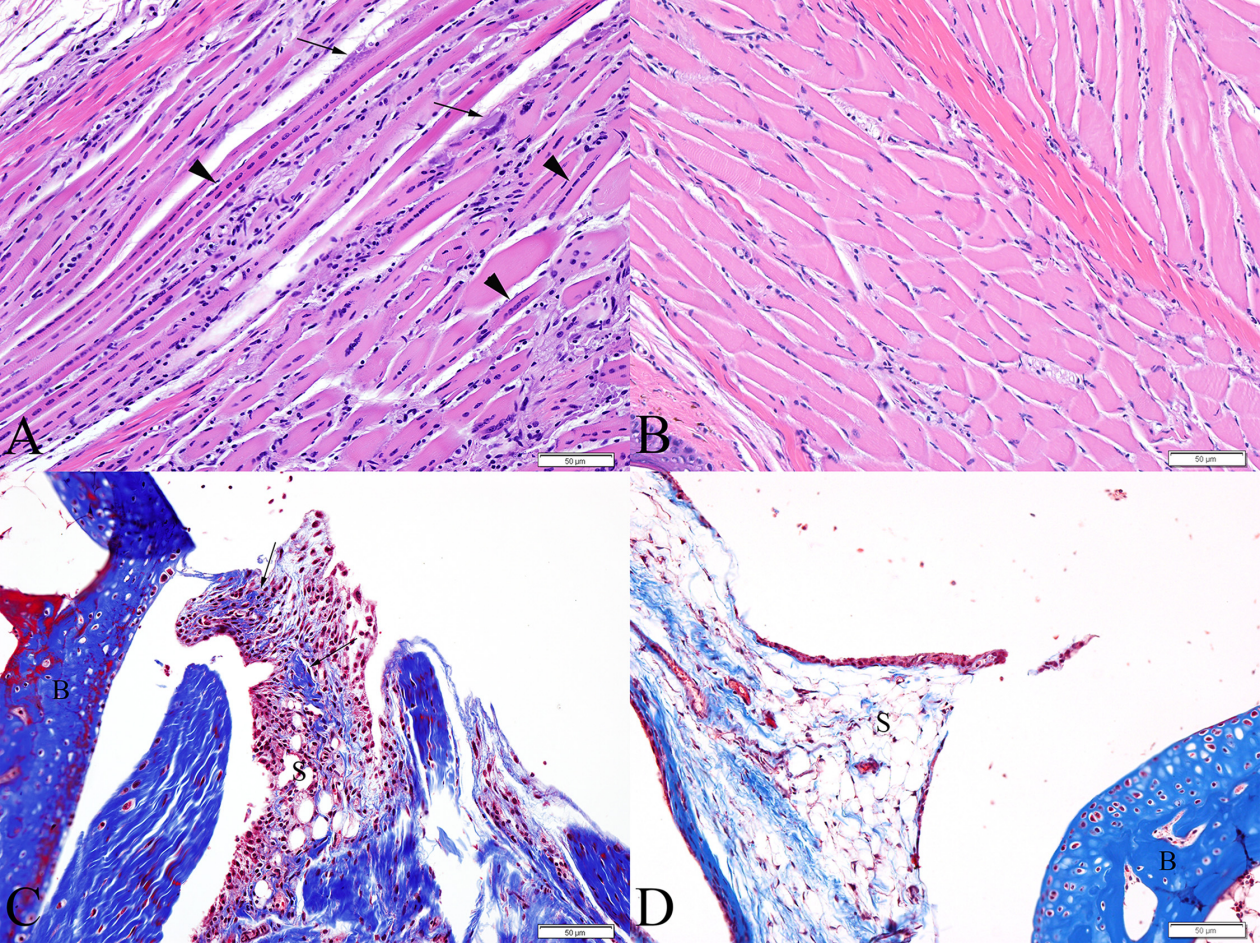
C57BL/6 mice sacrificed at 21 DPI. (A) Skeletal muscle exhibiting multifocal areas of ongoing necrosis (arrows) and extensive myofiber regeneration (arrowheads). (B) Skeletal muscle from a similar location of a normal PBS-inoculated control mouse. (C) Tibiotarsal joint demonstrating deposition of fibrous connective tissue within the synovium (arrows) (Masson’s trichrome stain). (D) Tibiotarsal from a PBS-inoculated control mouse demonstrating normal synovium (Masson’s trichrome stain). (S = synovium, B = bone)

**Fig 4:**
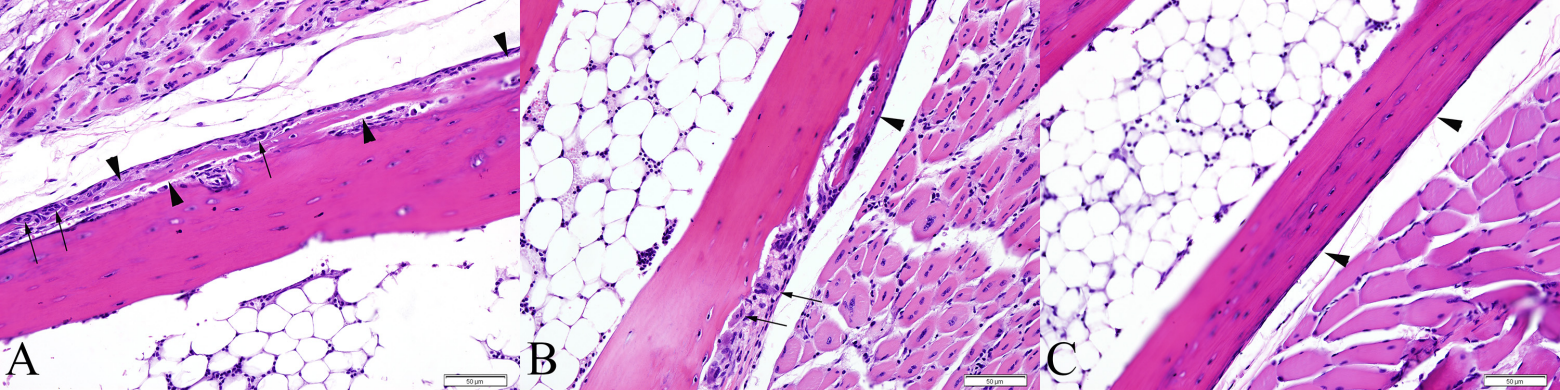
Metatarsal bones of C57BL/6J mice at 21 DPI exhibiting periosteal bone proliferation of woven bone. (A) Periosteal bone proliferation (arrowheads) and few large multinucleated cells within scalloped lacunae (osteoclasts, arrows) along the superficial margin of the periosteal new bone, just deep to the periosteal lining. (B) Periosteal bone proliferation (arrowhead) and osteoclasts (arrows) are apparent along the cortical surface of the bone. (C) Metatarsal bone from a PBS-inoculated control mouse demonstrating normal periosteum (arrowhead).

At 21 DPI, in the skeletal muscle, the necrosis was beginning to resolve and regeneration was more prominent and accompanied by some loose fibrous connective tissue replacing areas of myofiber dropout (Fig 3A). The subintimal mononuclear infiltrates within the synovium had increased and consisted predominantly of lymphocytes and plasma cells, consistent with a subacute lymphoplasmacytic synovitis affecting 4 of 4 mice (100%). Synoviocytes, when present, were often hypertrophic and trichrome stains demonstrated increased fibrous connective tissue within the synovium (Fig 3C). Two of 4 mice (50%), had foci of necrotic articular cartilage. Affecting 4 of 4 (100%) virally infected mice, there was extensive, moderate periostitis. In 2 of 4 (50%) of these mice, on the plantar surface of the diaphysis of the metatarsal bone, within areas of periostitis, there were focal regions of mild proliferation of immature woven bone beneath the periosteum (subperiosteal bone proliferation) (Fig 4A). These foci were limited to this location and not seen in other bones of the foot. Within these areas, lining the periosteal new bone, there were numerous elliptical cells with basophilic cytoplasm, a perinuclear clear zone and heterochromatic nuclei (consistent with activated osteoblasts). Occasionally, within the periosteum and associated with either periosteal new bone or the periosteal surface of adjacent cortical bone, there were few large, polygonal to round, multinucleated cells occasionally found within scalloped margins of bone (Howship’s lacunae), consistent with osteoclasts actively resorbing bone (Fig 4B). Bone marrow edema was apparent in 75% of mice.

In the contralateral feet of the majority of virally inoculated mice, minimal myocyte necrosis was occasionally observed at all timepoints. Additionally, minimal to mild synovitis and tenosynovitis were occasionally observed at all timepoints, predominantly associated with the distal phalanges. There were no additional significant findings in the contralateral feet of virally inoculated mice. None of the previously described lesions were present in either the inoculated or contralateral feet of control mice.

### IRF 3/7 ^-/- -/-^ mice

**Fig 5:**
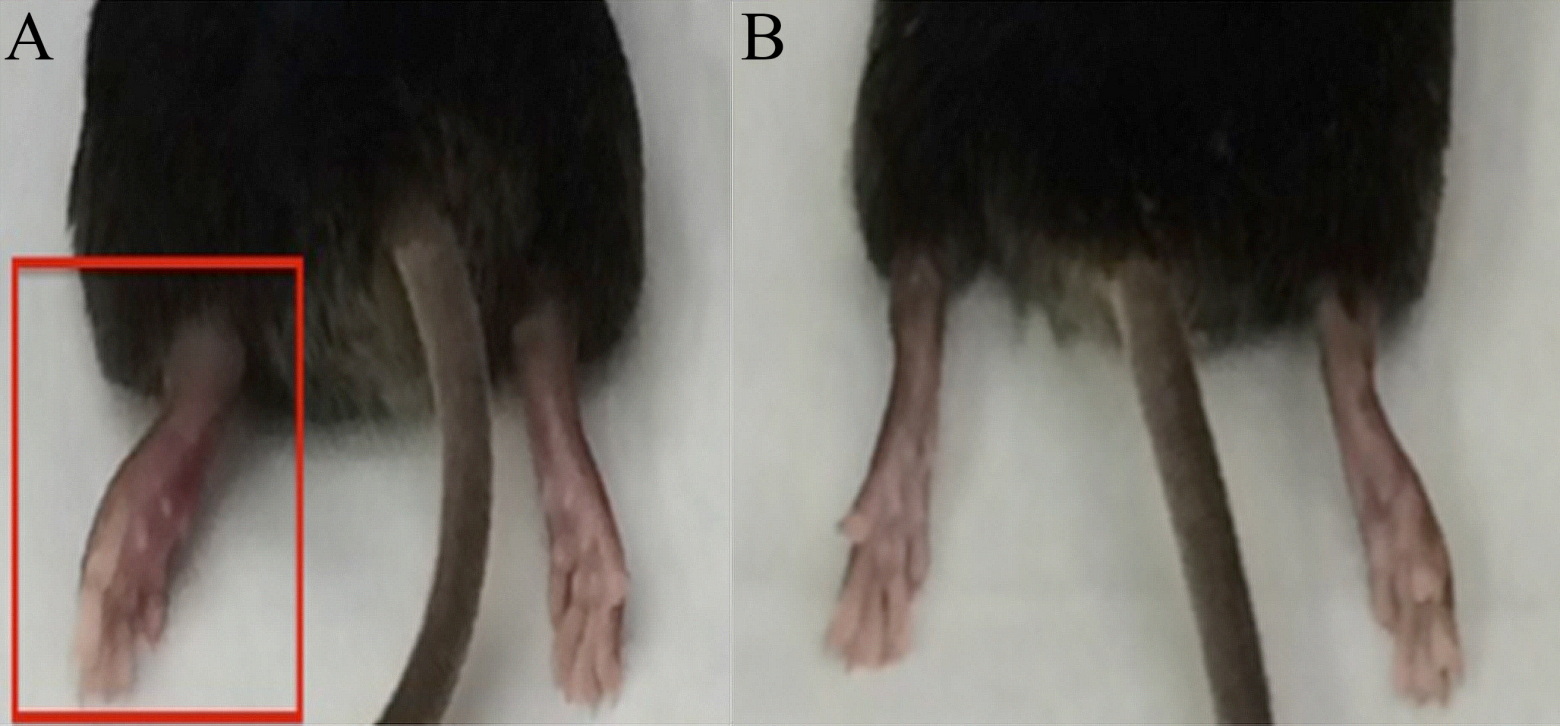
IRF 3/7 ^-/- -/-^ mice sacrificed at 5 DPI. (A) CHIKV inoculated mouse demonstrating significant swelling of the inoculated foot (left foot; red box). Mild hyperemia is apparent in the contralateral (right) foot. (B) PBS Sham inoculated mouse demonstrating no swelling associated with inoculation (left foot).

Similarly to what has been previously reported in this mouse model [41,42], grossly apparent swelling of the virus-inoculated feet was apparent by 2 DPI and reached its maximum at 4 DPI. While measureable swelling of the contralateral feet did not occur, mild hyperemia was apparent when compared to the PBS control mice (Fig 5). No additional significant gross findings were apparent.

**Fig 6:**
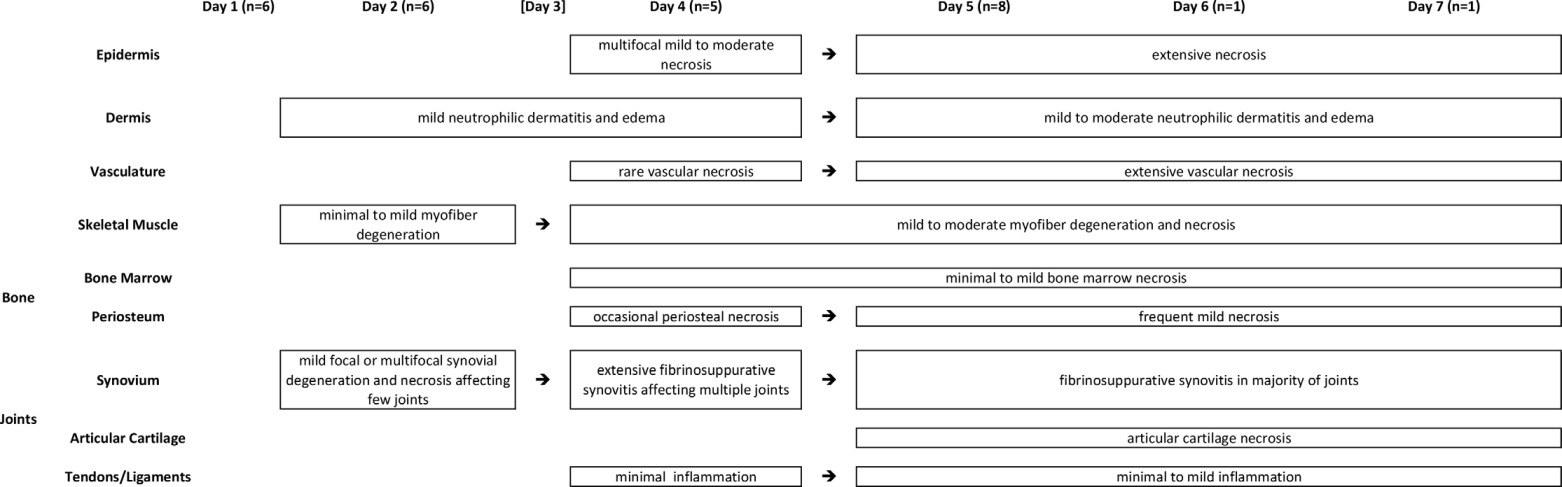
Progression of lesions in IRF 3/7 ^-/- -/-^ mice infected with chikungunya virus [mice were not evaluated on Day 3 post-infection].

Initial histopathologic evaluations of IRF 3/7 ^-/- -/-^ mice infected with CHIKV have been reported previously [41,42] and lesions similar to those described were also observed in the current experiment. These included multifocal epidermal necrosis, dermal edema, inflammation and hemorrhage, skeletal muscle necrosis, tenosynovitis, and fibrinonecrotizing vasculitis. Because these lesions have been previously described, the following results will consist of more detailed descriptions of some of these and descriptions of novel lesions. An overview of the various lesions and their time courses are presented in Fig 6.

At 2 DPI, initial evidence of minimal to mild skeletal muscle degeneration was present in 6 of 6 mice (100%). While a fibrinous synovitis has been previously reported in this mouse model [41,42], the full extent and progression of the joint disease has not been described. Initial synovial degeneration and necrosis was observed at 2 DPI in the inoculated feet in 100% mice, predominantly affecting the tarsal joints. Two of 6 mice (33%) exhibited variably severe regions of bone marrow necrosis characterized by locally extensive areas of eosinophilia, loss of cellular detail, accumulation of cellular debris, and presence of cells with pyknotic, karyolytic or karyorrhectic nuclei (apoptosis and necrosis).

**Fig 7:**
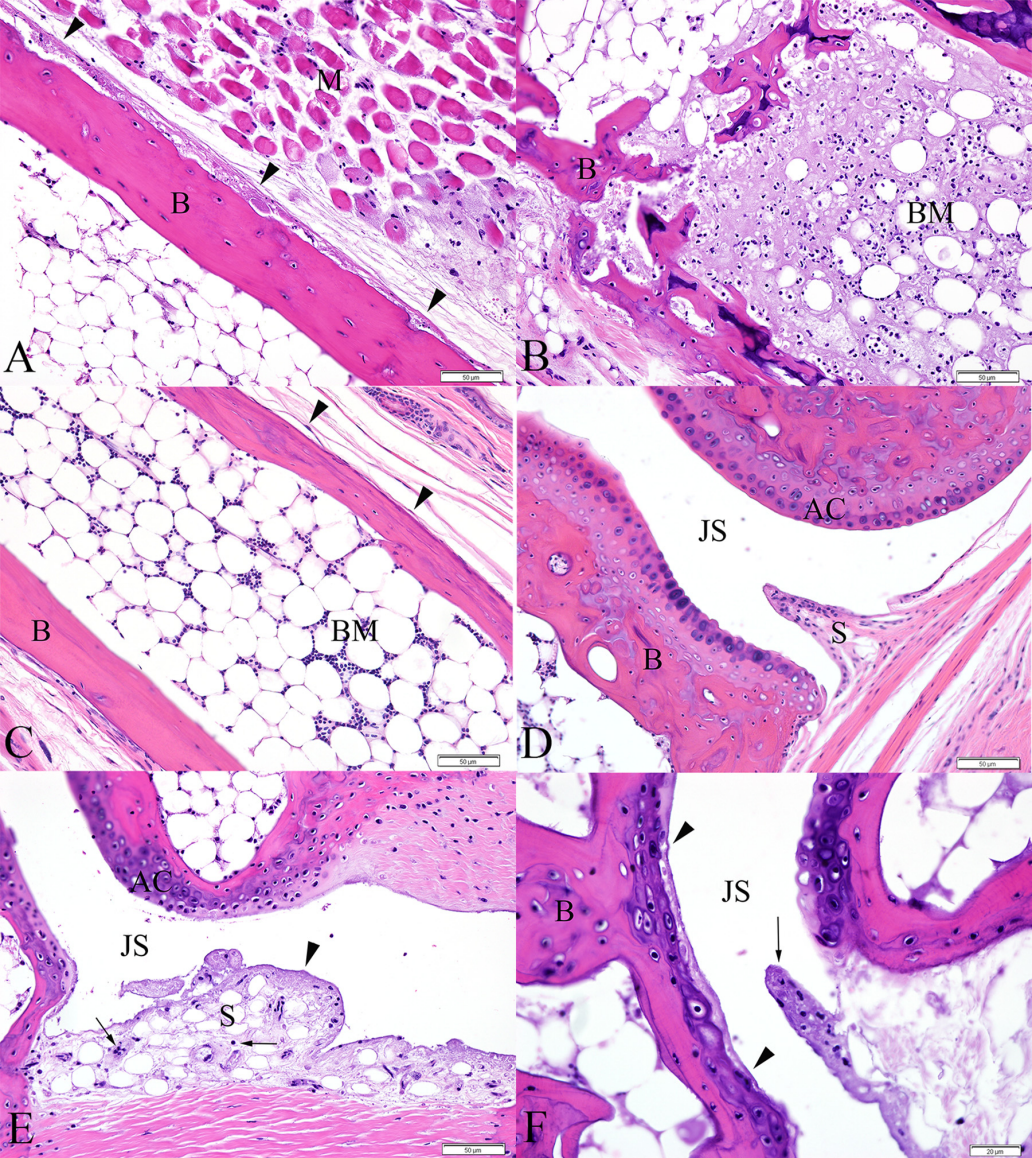
Metatarsal bones and tarsal joints from IRF 3/7 ^-/- -/-^ mice. (A) 4 DPI, periosteal necrosis of the metatarsal bone (arrowheads). The periosteum is slightly expanded by fibrin and cellular debris. (B) 4 DPI, distal aspect of metatarsal bone exhibiting extensive bone marrow necrosis. Normal bone marrow is replaced by fibrin, cellular debris, and necrotic and apoptotic cells. (C) Metatarsal bone from a PBS-inoculated control mouse demonstrating normal bone marrow and periosteum (arrowheads). (D) Synovium and articular cartilage from a normal PBS-inoculated control mouse. (E) 5DPI, fibrinous synovitis exhibiting loss of synoviocytes, accumulation of fibrin along the intima and within the superficial subintima (arrowhead), and infiltration of the subintima by few leukocytes (arrows). (F) 6 DPI, cartilage necrosis (bounded by arrowheads) is apparent in the articular cartilage of the metatarsal bone, adjacent to a region of fibrinous synovitis (arrow). (B = bone, M = skeletal muscle, BM = bone marrow, S = synovium, JS = joint space, AC = articular cartilage)

By 4 DPI, lesions of skeletal muscle degeneration progressed to necrosis in 5 of 5 mice (100%). Fibrinoid vasculitis affecting small and medium caliber vessels was apparent in 5 out of 5 mice (100%), though was not widespread. There was necrosis and loss of endothelial cells, transmural fibrinoid change of the vessel walls, and occasional leukocytoclasia. Inflammatory cells within the necrotic vessel walls were rare and consisted of degenerate leukocytes. The majority of joints throughout the foot, including the intertarsal, tarsometatarsal, metatarsophalangeal, and interphalangeal joints were affected in 100% of mice. In these cases, there was partial to complete loss of the superficial synoviocytes accompanied by degeneration and necrosis of any remaining synoviocytes. The subintima was infiltrated by rare neutrophils and mononuclear cells, and variably effaced and replaced by accumulations of fibrin and necrotic debris. Three of 5 mice exhibited focally extensive regions of minimal to mild periosteal necrosis characterized by loss of cellular detail and accumulation of small amounts of fibrin and necrotic debris (Fig 7A). Regions of bone marrow necrosis were present in 100% of mice (Fig 7B). Occasionally, regions of necrosis also contained numerous prominent vacuolated macrophages.

**Fig 8:**
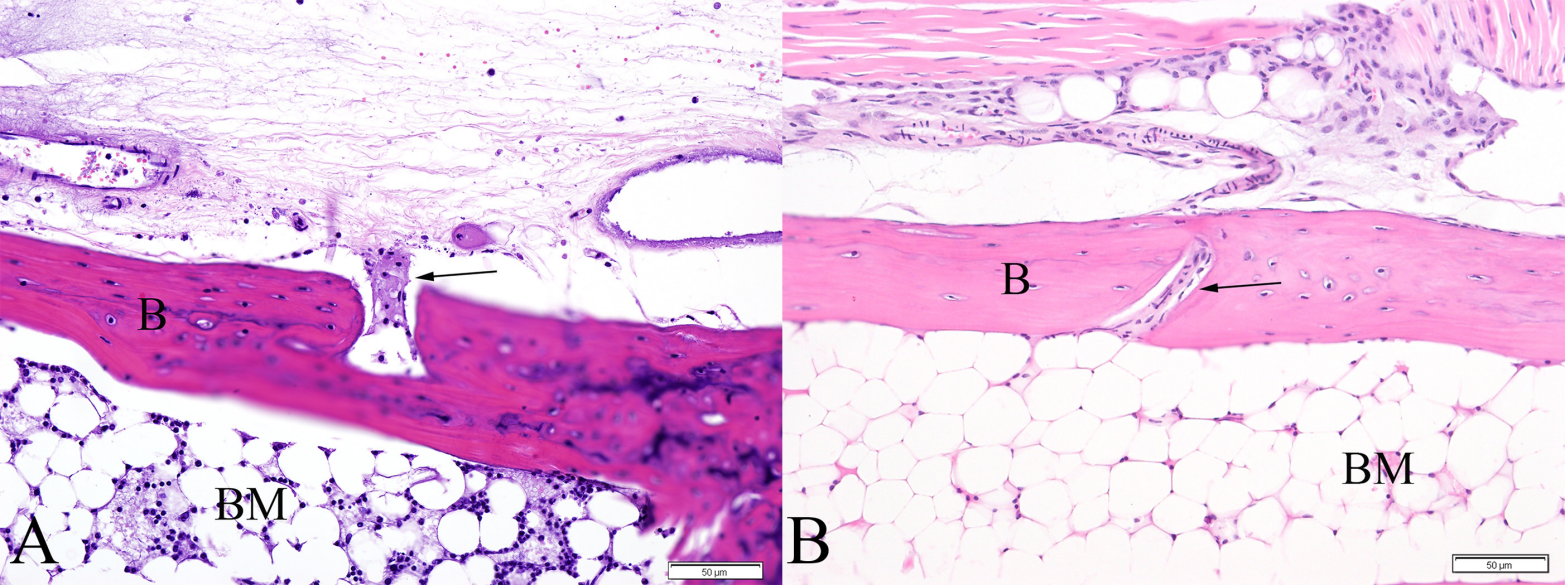
Metatarsal bones from IRF 3/7 ^-/- -/-^ mice. (A) 7 DPI, fibrinoid vasculitis affecting the mid-diaphyseal blood vessel of the metatarsal bone (arrow) associated with a region of bone marrow necrosis. (B) PBS sham inoculated mouse demonstrating normal mid-diaphyseal blood vessel of the metatarsal bone (arrow). (B = bone, M = skeletal muscle, BM = bone marrow)

By 5 DPI and later, 10 of 10 mice (100%) had extensive multifocal myocyte necrosis accompanied by minimal infiltration by rare neutrophils and macrophages. In 100% of mice, fibrinoid vasculitis was widespread and affected the majority of vessels, including occasional larger caliber vessels. Synovial lesions persisted at these timepoints in all mice (Fig 7E). Additionally, 3 out of 10 mice (30%) exhibited focal areas of cartilage necrosis located immediately adjacent to regions of fibrinous synovitis. The cartilage necrosis was characterized by decreased staining intensity of the cartilage matrix and presence of pale, swollen chondrocytes often exhibiting loss of cellular detail (necrosis) (Fig 7F). Eight out of 10 mice exhibited focally extensive regions of periosteal necrosis, particularly evident within the periosteum of the metatarsal bones and all mice (10/10) had multifocal and extensive regions of bone marrow necrosis. These lesions were present throughout the foot, often variably affecting numerous bones within the section extending from the tibia to the distal phalanges. In some instances, necrotic nutrient/mid-diaphyseal vessels were present near the most severe regions of bone marrow necrosis, consistent with ischemic necrosis of bone marrow (Fig 8A). Rarely, in the caudal aspect of the calcanei, regions of marrow necrosis extended into and affected the adjacent physeal cartilage.

In the contralateral feet of all virally inoculated mice, similar, though milder lesions of dermal edema and hemorrhage, fibrinonecrotizing vasculitis, fibrinous synovitis, and ischemic bone marrow necrosis were present at similar timepoints. None of the previously described lesions were present in either the inoculated or contralateral feet of control mice.

### Micro-computed tomography (μCT)

**Fig 9:**
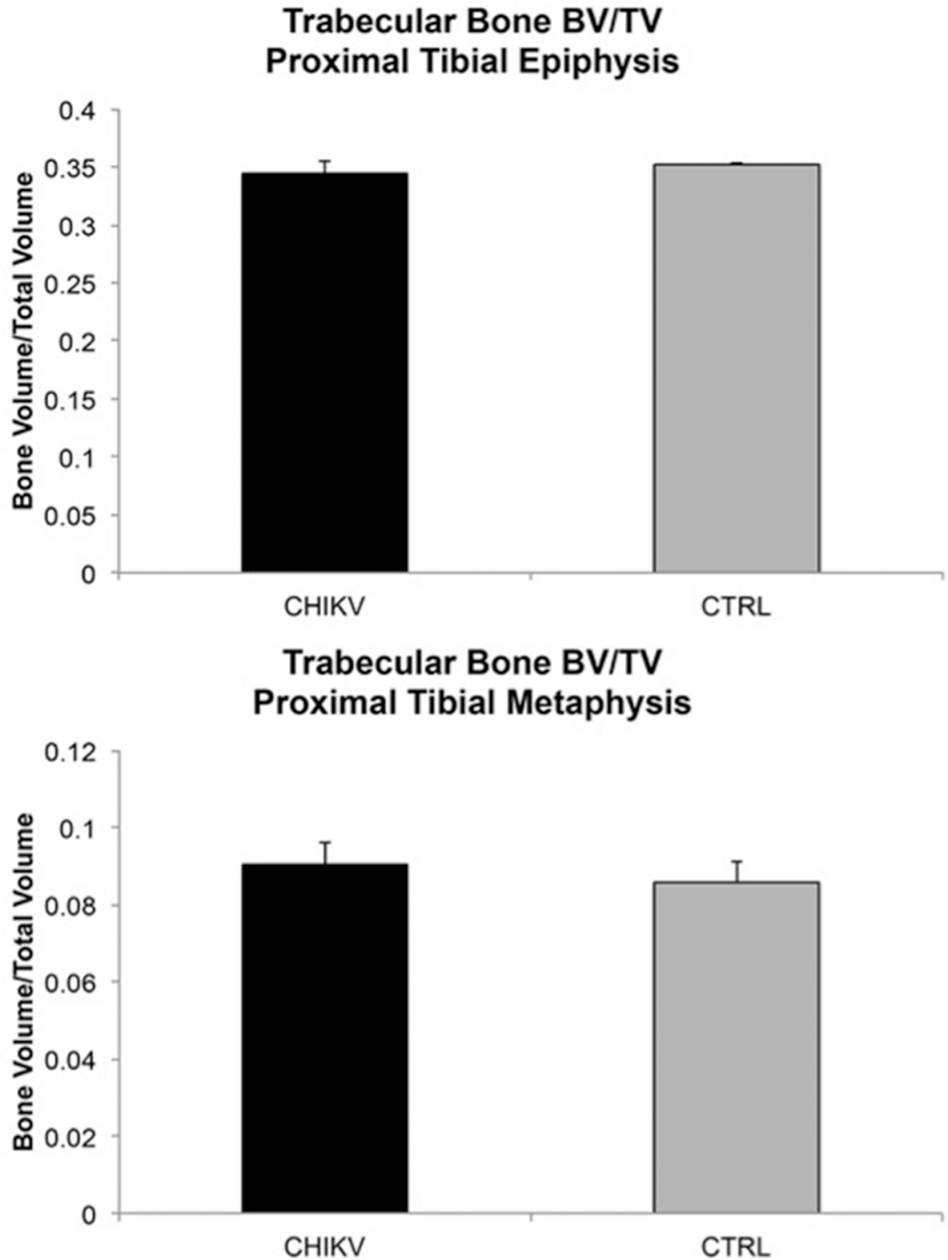
Bone volume/total volume in CHIKV and control mice. ROIs included the proximal epiphysis of the tibia and distal metaphysis of the femur. Mean ± SEM.

**Fig 10:**
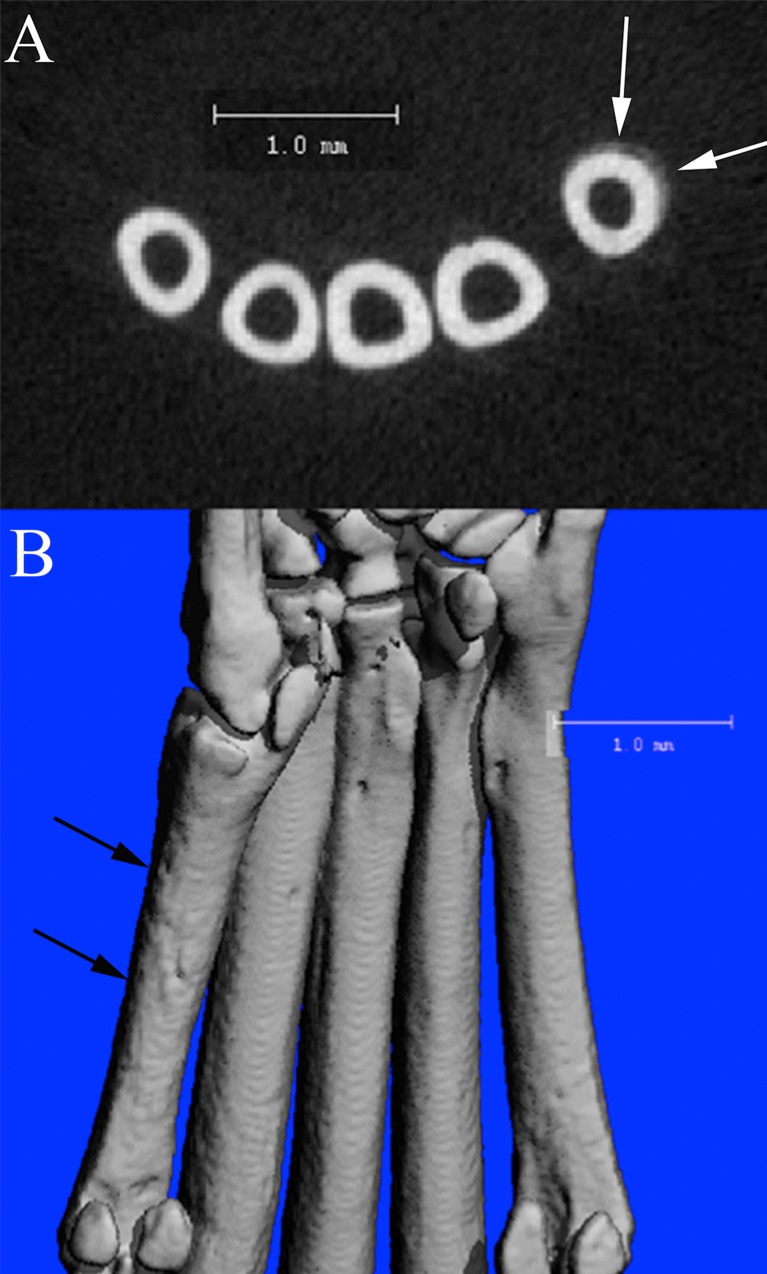
μCT scans of a CHIKV infected C57BL/6 mouse at 21 DPI. (A) 2D slice from μCT scan of the mid-metatarsal region demonstrating region of periosteal bone proliferation (arrows) on first metatarsal bone (Mt1). (B) Corresponding 3D reconstruction of the plantar metatarsal region of the same mouse, demonstrating the roughened periosteal surface on Mt1 (arrows) in the region of the proliferative lesion identified in A.

No changes in any trabecular bone parameters in wild-type C57BL/6J mice secondary to CHIKV infection were identified in either ROI evaluated (Fig 9). Within the wild-type C57BL/6J mice, 3 out of 4 mice (75%) at 21 DPI exhibited periosteal bone proliferation secondary to CHIKV infection, whereas this same lesion was not identified in any control mice. These lesions were readily visible on 2D μCT scans (Fig 10A), and 3D reconstructions of the region identified a roughened surface of the bone (Fig 10B). The first metatarsal bone of each mouse was most consistently and severely affected in all instances. The regions of periosteal bone proliferation affected between 17.48% and 46.10% of the entire bone length and the volume of the regions of periosteal bone ranged from 0.0079 mm^3^ to 0.0812 mm^3^. The density of these areas ranged from 458.6021 mg HA/ccm to 631.638 mg HA/ccm as compared to a density of the adjacent cortical bone between 922.7256 mg HA/ccm and 1013.2021 mg HA/ccm, consistent with the fact that immature woven bone is less dense than mature lamellar cortical bone.

**Fig 11:**
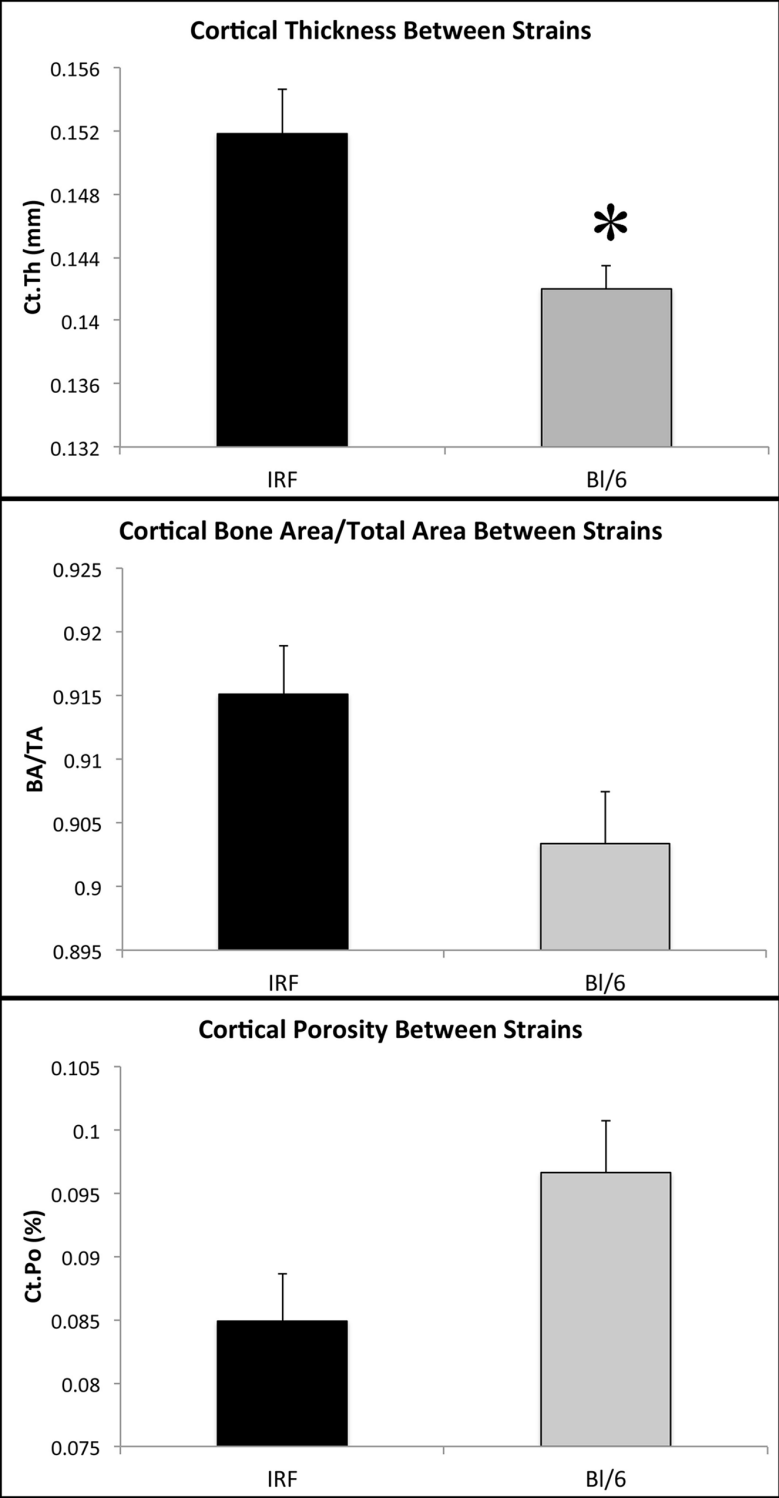
Comparison of cortical bone parameters in normal control IRF 3/7 ^-/- -/-^ and C57BL/6 mice. Specific parameters of interest include cortical bone thickness (Ct.Th), bone area/total area (BA/TA), and porosity (Ct.Po).

No changes were identified in trabecular bone between the two strains. However, several differences between the two strains were identified in cortical bone at the midshaft of the humerus (Fig 11). IRF 3/7 ^-/- -/-^ mice were found to have significantly higher Ct.Th (p = 0.010). Ct.Ar/Tt.Ar also trended towards being significantly higher (p = 0.050) in the IRF 3/7 ^-/- -/-^ mice, and Ct.Po trended towards being significantly lower (p = 0.050).

## Discussion

These experiments demonstrated novel pathologic lesions in the bones and joints of two established mouse models of chikungunya virus (CHIKV) infection. To our knowledge, this is the first description of cartilage necrosis associated with CHIKV infection in an adult immunocompetent wild-type animal model, though chondrocyte loss has been recently described in a CCR2 ^-/-^ mouse model [34]. Additionally, while the presence of CHIKV RNA has been demonstrated in the periosteum [42], the results from the current experiments are the first to demonstrate consistent periostitis and the presence of periosteal bone proliferation, as well as extensive ischemic bone marrow necrosis associated with CHIKV infection. These novel lesions offer new insights into the pathogenesis of acute, chronic, and fatal CHIKV-associated diseases.

It should be noted that different routes of viral inoculation were used in the two mouse models utilized in these experiments (i.e. intradermal for IRF 3/7 ^-/- -/-^ mice and subcutaneous for C57BL/6 mice). While route of inoculation can have some impact on immunological responses and disease progression, because early pathogenic mechanisms of viral infection and immune responses are not compared between the two mouse strains, this difference in technique is not considered to have significant impact on the importance of the novel lesions described herein. Experiments performed in our laboratory utilizing a subcutaneous route of infection in IRF 3/7 ^-/- -/-^ mice have demonstrated similar lesions at 5 DPI and later in both the ipsilateral and contralateral feet to those reported here in intradermally inoculated mice (data not shown). Therefore, the differences in disease manifestations between the two strains are considered to be predominantly a result of the specific mouse strains utilized and their different immunological responses to viral infection, rather than differences in inoculation technique [40–42,45]. As a result of dramatically diminished type 1 interferon response, the IRF mice develop a severe fatal form of disease and tissues demonstrate paucicellular, primarily necrotizing changes including severe, widespread fibrinoid vascular necrosis resulting in severe edema and hemorrhage [41,42]. This is in contrast to a histiocytic and lymphocytic inflammatory response which occurs in the C57BL/6 mice, resulting in a more inflammatory condition and a milder and more protracted from of disease [40,45].

### Cartilage–IRF 3/7 ^-/- -/-^ and C57BL/6 mice

Early evidence of articular cartilage necrosis associated with CHIKV infection is significant because chondrocytes have limited ability to replicate and repair, therefore this damage is often considered irreversible [46–48]. Attempts at cartilage repair consist of proliferation of fibrocartilaginous tissue, which will gradually degenerate over time due to differences in biomechanical properties when compared to normal articular hyaline cartilage [48]. Therefore, if the initial damage to the articular cartilage is severe enough, it is often progressive [46]. Even in the absence of ongoing infection and/or inflammation, these types of lesions can progress as degenerative changes, similar to those seen in osteoarthritis [49].

### Periostitis and periosteal new bone–C57BL/6 mice

As previously mentioned, this report is the first to describe periostitis and periosteal bone proliferation associated with CHIKV infection in an animal model. Periostitis has been occasionally described in human cases of CHIKV-associated disease, often affecting the ankles and wrists, and associated with metacarpophalangeal and proximal interphalangeal stiffness [26,50]. Additionally, periostitis accompanied by periosteal bone proliferation associated with tenosynovitis was demonstrated via radiography in a patient with post-CHIKV infection disease [37].

The presence of periostitis with periosteal bone proliferation associated with CHIKV infection may shed some light on the mechanisms involved in the severe polyarthralgia that occurs with CHIKV infection both acutely and chronically. The periosteum contains numerous sympathetic nerves and thus irritation and inflammation result in significant sensation of pain [51]. Because periostitis often manifests as painful limbs, particularly as polyarthralgia/polyarthritis when affecting distal extremities, it can go unrecognized and be misdiagnosed as a myopathy or joint disease, such as rheumatoid arthritis [1]. Additionally, while periosteal bone proliferation can be a reversible process [1,52], some areas can persist. These can remain clinically inapparent, but can also interfere with adjacent tendon or ligament function resulting in clinical manifestations such as stiffness or discomfort [52]. The current experiments suggest that pain associated with both acute and chronic cases of CHIKV-associated arthralgia may, at least in part, be a result of development of periostitis and periosteal bone proliferation.

### Ischemic necrosis of bone marrow–IRF 3/7 ^-/- -/-^ mice

Another lesion demonstrated in these experiments that may be relevant in regard to severe and fatal manifestations of CHIKV-associated disease is widespread ischemic necrosis of bone marrow seen in the IRF 3/7 ^-/- -/-^ mice. Clinically, regardless of the inciting cause, bone marrow necrosis often presents as bone pain (75% of cases) and fever (68.5% cases) in human patients [53,54]. Ischemic bone marrow necrosis can progress to true osteonecrosis that ultimately can result in bone destruction and joint pain [55].

It is currently unknown whether ischemic bone marrow necrosis represents a significant component of severe CHIKV disease in people. This manifestation of disease has not been described in the literature in humans, possibly because bone marrow biopsies and extensive autopsies including evaluation of numerous regions of bone marrow are not commonly performed in severe and fatal cases and/or results of these autopsies may not be reported in the literature. Virus-associated vasculitis has been reported in cases of CHIKV infection in people, therefore, ischemic necrosis of tissues including bone marrow is potentially possible [56–58].

### μCT

Results of these experiments that are of additional interest include the lack of significant differences in measurements of bone volumes in the virally infected C57BL/6J mice as compared to the negative control mice. It has recently been reported that infection with CHIKV resulted in significant decreases in various bone parameters, including trabecular bone mass and bone volume fraction at 3 DPI, as well as increased osteoclastic activity [43]. However, these findings were not recapitulated in the current experiments. The most likely reason for this is associated with the ages of the animals used in the respective experiments.

In the current experiment, adult, skeletally mature C57BL/6J mice were utilized, as compared to juvenile (25 day old), skeletally immature C57BL/6 mice used in the previously reported experiments [43]. Bone growth, modeling and remodeling dynamics are quite different in juveniles vs. adults and thus alterations in any of these processes can also be very different depending on the age of the individual. In experiments utilizing the juvenile model, the authors stated that there was a decreased width of the growth plates in affected mice, demonstrating potential alterations in bone growth in addition to increased osteoclastic bone resorption [43]. Therefore, the results in the immature mice, in regard to bone dynamics, may not be directly applicable to chikungunya virus disease in adult humans as active bone growth is no longer occurring. However, it is unclear if these alterations might be similar to those occurring in neonatal and juvenile cases of CHIKV infection.

The results of the previous experiments, in conjunction with those of the current experiments suggest it is possible that CHIKV affects bone growth and growth plate function in juveniles and that there is less of an effect on the mechanics of bone remodeling in an adult animal model. Therefore the current experiments may more closely replicate disease in adult patients. While decreased bone volumes were not demonstrated in the current experiments, osteoclastic bone resorption appears to still be a component of disease. Active osteoclasts were demonstrated in the areas of periosteal bone proliferation, affecting both the new woven bone as well as the adjacent existing lamellar cortical bone.

In addition to the analyses performed in regard to CHIKV infection, the normal control IRF 3/7 ^-/- -/-^ and wild-type C57BL/6J mice were compared for differences in normal bone structure. The results indicated that there were significant and near-significant differences in the cortical bone between these two strains. This indicates that a thorough analysis and understanding of the normal bone morphology of inbred strains of mice is essential when comparing bony changes, as this may impact results identified or the underlying mechanisms for changes. Other studies have identified significant differences in normal bone morphology and remodeling processes among popular mouse strains, including in both trabecular and cortical bone [59–65]. The mechanism(s) involved in the differences in cortical bone between the two strains utilized in the current experiments is unknown, but indicate potential differences in bone growth, modeling and remodeling. Therefore appropriate choice of mouse strain utilized in studies of bone and joint pathology is essential, and a thorough evaluation and characterization of skeletal structure is necessary in all experiments.

A potential drawback of this study is that virus and/or viral antigen was not demonstrated within the tissue sections. Numerous attempts were made to develop an immunohistochemistry protocol using a commercially available anti-chikungunya virus mouse antibody (LifeSpan BioSciences, Inc., Seattle, WA) and Mouse on Mouse peroxidase and fluorescein kits (Vector Laboratories, Burlingame, CA) (data not shown). Unfortunately, background staining or fluorescence including non-specific binding of the secondary antibody was observed in these attempts and could not be diminished to a level at which the authors could be confident in the interpretation of results. However, because established mouse models were utilized in these experiments and lesions were consistent within virally inoculated animals while absent in control animals, the novel lesions described herein are still considered significant.

## Conclusions

In closing, this study has demonstrated numerous novel findings in bone and joints that may aid in further determination of the pathogenesis of acute, chronic and severe forms of CHIKV-associated disease. The identification of periostitis and periosteal bone proliferation demonstrates a component of disease manifestation that may be under-recognized in human cases. Potentially progressive lesions such as cartilage necrosis and periosteal bone proliferation, and disease manifestations resulting in permanent tissue alterations, such as fibrosis within skeletal muscle and synovium, have demonstrated the utility of these mouse models in future studies focusing on chronic CHIKV-associated disease. These experiments also illustrate the importance of advanced imaging techniques in evaluation of diseases involving bones and joints, as without the use of μCT, the presence of some bony lesions may have been overlooked and certainly the extent of these lesions would not have been fully appreciated.

Currently, the mechanisms involved in the development of cartilage necrosis, periostitis and periosteal bone formation, and ischemic bone marrow necrosis are unknown. Experiments examining the local environment within the joint, including alterations in regulation of cytokine and matrix metalloproteinase production, may help elucidate the mechanisms involved in CHIKV-associated cartilage necrosis. A definitive reason for the consistently affected location of the periosteal bone proliferation is currently unknown, though possibilities could include anatomical structure of the vasculature within this region or variable severity of inflammation in surrounding tissues. Further experiments are required for clarification of the factors involved in periostitis and periosteal bone proliferation and to determine the significance of ischemic bone marrow necrosis associated with CHIKV infection. Additionally, the incorporation of evaluation via radiographs, CT and MRI into clinical studies of patients with acute and chronic CHIKV-associated arthralgia will greatly enhance the current understanding of disease pathogenesis.
